# Supplementary material for: A quantum chemical approach representing a new perspective concerning agonist and antagonist drugs in the context of schizophrenia and Parkinson’s disease
Source: PLoS One. 2019 Dec 12;14(12):e0224691. doi: 10.1371/journal.pone.0224691 (PMC6907805; doi:10.1371/journal.pone.0224691)
Supplement: S1 Data — (PDF) [file pone.0224691.s001.pdf]

A quantum chemical approach representing a new perspective concerning agonist and antagonist drugs in the context of schizophrenia and Parkinson's disease

Ana Martínez, Ilich A. Ibarra and Rubicelia Vargas

Supporting Information. Cartesian coordinates of the optimized structures

Dopamine

Input orientation:

Center Number	Atomic Number	Atomic Type	Coordinates (Angstroms) X	Y	Z
1	8	0	-2.230119	1.929383	0.083198
2	8	0	-3.338823	-0.506060	0.441460
3	7	0	4.404831	0.260301	0.424525
4	6	0	2.176377	-0.014399	-0.706995
5	6	0	0.709491	-0.173500	-0.450821
6	6	0	2.962927	0.123869	0.590801
7	6	0	-0.110106	0.944534	-0.322296
8	6	0	0.150371	-1.432662	-0.268236
9	6	0	-1.453401	0.810419	-0.025724
10	6	0	-1.197597	-1.574669	0.030601
11	6	0	-2.001919	-0.457670	0.152472
12	1	0	2.558513	-0.883521	-1.257698
13	1	0	2.362245	0.871806	-1.327377
14	1	0	2.762407	-0.749437	1.222758
15	1	0	2.588139	0.993434	1.143591
16	1	0	0.296343	1.944736	-0.458220
17	1	0	0.776500	-2.316331	-0.363189
18	1	0	-1.641022	-2.557718	0.170283
19	1	0	4.585073	1.067996	-0.168850
20	1	0	4.743142	-0.540200	-0.106507
21	1	0	-3.136082	1.664120	0.296827
22	1	0	-3.616989	-1.424832	0.550195

Ropinirole

Input orientation:

Center Number	Atomic Number	Atomic Type	Coordinates (Angstroms) X	Y	Z

1	8	0	-3.380828	3.163823	-0.510254
2	7	0	2.622027	-0.153783	0.131491
3	7	0	-3.877456	0.938868	-0.243566
4	6	0	1.255199	-0.638122	-0.051479
5	6	0	0.405875	-0.564306	1.209521
6	6	0	-1.022325	-0.889907	0.907253
7	6	0	2.624713	1.310309	0.080559
8	6	0	3.471944	-0.680852	-0.938016
9	6	0	-1.895939	0.089356	0.473801
10	6	0	-1.701205	1.554233	0.261086
11	6	0	-3.214617	-0.226745	0.157081
12	6	0	3.856073	1.961273	0.673419
13	6	0	3.893253	-2.123203	-0.755437
14	6	0	-1.512325	-2.195746	1.001889
15	6	0	-3.057761	2.024195	-0.210081
16	6	0	-3.710011	-1.509785	0.251229
17	6	0	-2.827654	-2.496518	0.681345
18	6	0	3.799805	3.468346	0.525820
19	6	0	4.744120	-2.593910	-1.917522
20	1	0	0.760720	-0.080692	-0.871625
21	1	0	1.287077	-1.684669	-0.372683
22	1	0	0.468692	0.438004	1.649474
23	1	0	0.804206	-1.268362	1.948635
24	1	0	2.490982	1.648676	-0.966964
25	1	0	1.750617	1.681270	0.629224
26	1	0	2.963348	-0.561682	-1.916019
27	1	0	4.378172	-0.067658	-0.992913
28	1	0	-1.422231	2.100289	1.169499
29	1	0	-0.947229	1.797107	-0.496697
30	1	0	3.927347	1.689122	1.733886
31	1	0	4.763879	1.577086	0.192824
32	1	0	4.453998	-2.214456	0.183125
33	1	0	3.013780	-2.770731	-0.655966
34	1	0	-0.843522	-2.984005	1.339490
35	1	0	-4.849090	0.987346	-0.524916
36	1	0	-4.741082	-1.737858	0.001560
37	1	0	-3.178776	-3.520204	0.769403
38	1	0	3.764579	3.760117	-0.529624
39	1	0	2.906246	3.878700	1.009696
40	1	0	4.671800	3.951778	0.974691
41	1	0	4.189418	-2.539152	-2.860686
42	1	0	5.639757	-1.972070	-2.026699
43	1	0	5.072528	-3.628481	-1.785654

PD 128,907

Input orientation:

Center Number	Atomic Number	Atomic Type	Coordinates (Angstroms) X	Y	Z
1	8	0	0.054119	-1.988451	-0.132693
2	8	0	-0.502940	2.102571	0.050933
3	8	0	-4.925849	-1.167746	-0.222822
4	7	0	2.335653	-0.295367	0.340851
5	6	0	1.019306	0.230304	-0.018330
6	6	0	-0.067444	-0.714184	0.464773
7	6	0	2.443231	-1.640987	-0.228692
8	6	0	0.709967	1.582708	0.585746
9	6	0	-1.420773	-0.137373	0.183500
10	6	0	3.394767	0.553201	-0.212000
11	6	0	1.318008	-2.535908	0.209928
12	6	0	-1.563928	1.234991	0.008816
13	6	0	4.804020	0.086909	0.085899
14	6	0	-2.549460	-0.946436	0.110229
15	6	0	-2.813483	1.782008	-0.247216
16	6	0	-3.793689	-0.398843	-0.142995
17	6	0	5.811479	1.133109	-0.347991
18	6	0	-3.927787	0.970658	-0.325544
19	1	0	0.931387	0.318116	-1.121346
20	1	0	0.057747	-0.833769	1.557319
21	1	0	3.382668	-2.101000	0.086984
22	1	0	2.454088	-1.582460	-1.334092
23	1	0	0.623203	1.497899	1.679189
24	1	0	1.466802	2.332313	0.356851
25	1	0	3.266268	0.645787	-1.308948
26	1	0	3.285273	1.560052	0.202452
27	1	0	1.392658	-3.505401	-0.287572
28	1	0	1.358966	-2.698468	1.297796
29	1	0	4.903963	-0.110749	1.160751
30	1	0	5.019291	-0.855937	-0.428737
31	1	0	-2.453761	-2.020538	0.246912
32	1	0	-2.897916	2.855470	-0.386217
33	1	0	5.715766	1.349748	-1.417866
34	1	0	5.663201	2.074112	0.192739
35	1	0	6.837940	0.802357	-0.168118
36	1	0	-4.908781	1.390577	-0.525333
37	1	0	-4.692462	-2.093472	-0.076556

7-OH-DPAT

Input orientation:

Center Number	Atomic Number	Atomic Type	Coordinates (Angstroms) X	Y	Z
1	8	0	4.935726	1.665098	-0.028781
2	7	0	-2.153902	-0.088066	0.572209
3	6	0	-0.815567	-0.425257	0.053714
4	6	0	-0.393864	-1.830614	0.463305
5	6	0	0.263167	0.529382	0.540578
6	6	0	0.855012	-2.250699	-0.287588
7	6	0	1.654968	0.094797	0.166436
8	6	0	1.942342	-1.218084	-0.209003
9	6	0	-3.174836	-0.826170	-0.174478
10	6	0	-2.443345	1.348890	0.571768
11	6	0	-4.591546	-0.661532	0.335722
12	6	0	-2.508838	2.053810	-0.776300
13	6	0	2.678522	1.036535	0.214911
14	6	0	3.256739	-1.547999	-0.527471
15	6	0	3.978369	0.687625	-0.102133
16	6	0	4.276803	-0.615335	-0.479341
17	6	0	-5.528429	-1.623260	-0.368219
18	6	0	-2.575957	3.554805	-0.581472
19	1	0	-0.825340	-0.387052	-1.055692
20	1	0	-1.186423	-2.560902	0.283568
21	1	0	-0.200445	-1.827868	1.545879
22	1	0	0.196160	0.604889	1.636853
23	1	0	0.110217	1.543189	0.155459
24	1	0	0.597528	-2.427664	-1.342219
25	1	0	1.227655	-3.208438	0.093246
26	1	0	-3.146662	-0.561908	-1.249182
27	1	0	-2.931033	-1.891997	-0.128079
28	1	0	-3.397153	1.499397	1.091194
29	1	0	-1.701248	1.851820	1.199577
30	1	0	-4.613860	-0.839150	1.418720
31	1	0	-4.944540	0.364612	0.180676
32	1	0	-1.631764	1.796941	-1.383571
33	1	0	-3.384635	1.713742	-1.342351
34	1	0	2.460982	2.062894	0.503420
35	1	0	3.485570	-2.571002	-0.819342
36	1	0	5.299604	-0.886366	-0.728552
37	1	0	-5.507478	-1.468905	-1.452997
38	1	0	-5.243746	-2.664029	-0.178726
39	1	0	-6.562586	-1.495325	-0.036559
40	1	0	-3.433144	3.832252	0.043086
41	1	0	-1.672957	3.923891	-0.081032

42	1	0	-2.671992	4.088379	-1.531348
43	1	0	5.794923	1.294873	-0.269185

Rotigotine

Input orientation:

Center Number	Atomic Number	Atomic Type	Coordinates (Angstroms) X	Y	Z
1	16	0	-5.184766	-0.192324	0.393294
2	8	0	4.238542	-2.768929	0.121527
3	7	0	-0.735355	1.267261	-0.738759
4	6	0	0.450200	0.518807	-0.284906
5	6	0	0.707038	-0.733344	-1.106584
6	6	0	1.704669	1.379259	-0.348284
7	6	0	1.816427	-1.567183	-0.492324
8	6	0	2.974198	0.627489	-0.055857
9	6	0	3.025313	-0.761759	-0.127149
10	6	0	-1.932379	0.435814	-0.823532
11	6	0	-0.951590	2.445750	0.109587
12	6	0	-2.375082	-0.261820	0.467740
13	6	0	-2.215828	3.231840	-0.171066
14	6	0	4.116027	1.348715	0.293174
15	6	0	4.230788	-1.401119	0.172155
16	6	0	5.304191	0.697932	0.566502
17	6	0	5.367109	-0.686250	0.509473
18	6	0	-3.651048	-0.994762	0.260787
19	6	0	-2.200856	4.546965	0.583228
20	6	0	-3.825831	-2.290900	-0.119423
21	6	0	-5.187243	-2.651156	-0.299673
22	6	0	-6.040579	-1.622122	-0.059256
23	1	0	0.312741	0.211554	0.772848
24	1	0	0.977235	-0.430846	-2.128830
25	1	0	-0.183077	-1.362127	-1.184667
26	1	0	1.650449	2.216545	0.353216
27	1	0	1.768967	1.824398	-1.353033
28	1	0	2.099592	-2.372237	-1.179230
29	1	0	1.434151	-2.072649	0.407701
30	1	0	-2.754218	1.059066	-1.187464
31	1	0	-1.792738	-0.324795	-1.597600
32	1	0	-0.931838	2.169683	1.182212
33	1	0	-0.110688	3.130634	-0.038632
34	1	0	-2.475661	0.470191	1.278140
35	1	0	-1.613597	-0.984448	0.785170
36	1	0	-2.302463	3.418977	-1.249248

37	1	0	-3.106711	2.661629	0.119803
38	1	0	4.058952	2.432954	0.351583
39	1	0	6.189880	1.265524	0.835542
40	1	0	6.288424	-1.219901	0.730169
41	1	0	-2.097233	4.380387	1.661390
42	1	0	-1.363019	5.177020	0.265534
43	1	0	-3.123034	5.112522	0.423536
44	1	0	5.123830	-3.089097	0.339568
45	1	0	-2.989559	-2.969784	-0.260759
46	1	0	-5.519005	-3.641338	-0.592093
47	1	0	-7.120928	-1.613729	-0.110709

----

cis-8-OH-PBZI

Input orientation:

Center Number	Atomic Number	Atomic Type	Coordinates (Angstroms) X	Y	Z
1	8	0	4.615391	1.676478	-0.287351
2	7	0	-2.284060	0.238143	-0.266292
3	6	0	-1.158309	-0.452973	0.390125
4	6	0	-0.047154	0.608992	0.553528
5	6	0	-0.560737	1.800895	-0.253947
6	6	0	-0.713425	-1.681225	-0.374670
7	6	0	-2.053335	1.667011	-0.079308
8	6	0	1.349279	0.150483	0.234438
9	6	0	0.559706	-2.231156	0.238796
10	6	0	1.653943	-1.210706	0.130919
11	6	0	-3.562409	-0.212685	0.254934
12	6	0	2.362786	1.092680	0.089386
13	6	0	-4.763868	0.378921	-0.448773
14	6	0	2.969145	-1.586543	-0.114960
15	6	0	3.667635	0.695970	-0.151556
16	6	0	3.980344	-0.651341	-0.252781
17	6	0	-6.054012	-0.211872	0.082714
18	1	0	-1.469219	-0.782186	1.398185
19	1	0	-0.048612	0.918512	1.608349
20	1	0	-0.289086	1.691043	-1.311768
21	1	0	-0.173738	2.759473	0.099619
22	1	0	-0.519643	-1.406071	-1.420937
23	1	0	-1.513994	-2.429736	-0.376103
24	1	0	-2.347801	1.987868	0.940135
25	1	0	-2.630961	2.260890	-0.793221
26	1	0	0.870579	-3.158229	-0.253321
27	1	0	0.372965	-2.486303	1.292775

28	1	0	-3.600775	-1.306793	0.165585
29	1	0	-3.632174	0.014132	1.339568
30	1	0	2.146497	2.155580	0.169148
31	1	0	-4.774465	1.467833	-0.316838
32	1	0	-4.680989	0.195615	-1.527702
33	1	0	3.205542	-2.644157	-0.209663
34	1	0	5.005489	-0.954901	-0.449496
35	1	0	-6.085506	-1.295452	-0.076565
36	1	0	-6.154401	-0.035075	1.159375
37	1	0	-6.930390	0.221647	-0.406956
38	1	0	5.475463	1.271838	-0.461670

7-OH-PIPAT

Input orientation:

Center Number	Atomic Number	Atomic Type	Coordinates (Angstroms) X	Y	Z
1	53	0	-3.549992	-3.668704	1.876499
2	8	0	5.158512	1.460708	1.061506
3	7	0	-1.921802	0.662999	-0.850849
4	6	0	-0.656960	0.013398	-0.456570
5	6	0	-0.183540	-1.013863	-1.473764
6	6	0	0.459653	1.029483	-0.259149
7	6	0	0.980627	-1.804790	-0.911027
8	6	0	1.819800	0.411315	-0.073552
9	6	0	2.076807	-0.919806	-0.395853
10	6	0	-2.288912	1.703024	0.119456
11	6	0	-3.001742	-0.318951	-1.020400
12	6	0	2.850481	1.210188	0.418192
13	6	0	-3.641919	2.351758	-0.091302
14	6	0	3.367784	-1.413848	-0.222484
15	6	0	4.124968	0.700144	0.582793
16	6	0	4.393116	-0.623487	0.259532
17	6	0	-3.289931	-1.150969	0.194163
18	6	0	-3.780180	3.576839	0.790795
19	6	0	-3.203506	-2.467210	0.185753
20	1	0	-0.802502	-0.511695	0.510347
21	1	0	0.119410	-0.487066	-2.390468
22	1	0	-0.976854	-1.710557	-1.751317
23	1	0	0.263877	1.671330	0.604873
24	1	0	0.487626	1.695426	-1.135569
25	1	0	1.382217	-2.486576	-1.668891
26	1	0	0.610147	-2.446110	-0.096263
27	1	0	-2.235987	1.315990	1.155757
28	1	0	-1.538867	2.497397	0.057395

29	1	0	-2.773207	-0.973253	-1.864641
30	1	0	-3.905642	0.224702	-1.308826
31	1	0	2.653524	2.249844	0.677347
32	1	0	-3.756637	2.633355	-1.146172
33	1	0	-4.453398	1.651114	0.136555
34	1	0	3.570825	-2.451646	-0.478964
35	1	0	5.397875	-1.014253	0.389649
36	1	0	-3.557703	-0.624416	1.109721
37	1	0	-3.643846	3.317225	1.846746
38	1	0	-3.029520	4.333403	0.538356
39	1	0	-4.766477	4.037667	0.688649
40	1	0	-2.932714	-3.044745	-0.693601
41	1	0	4.838394	2.352337	1.250878

----

pramipexole

Input orientation:

Center Number	Atomic Number	Atomic Type	Coordinates (Angstroms) X	Y	Z
1	16	0	2.298815	-1.778270	0.384254
2	7	0	-2.422574	0.659028	0.403272
3	7	0	3.156713	0.565835	-0.278884
4	7	0	4.864424	-1.048860	-0.122898
5	6	0	-1.037620	0.612161	-0.050528
6	6	0	-0.315980	1.886480	0.350481
7	6	0	-0.315153	-0.604710	0.519783
8	6	0	1.077132	1.959231	-0.253865
9	6	0	1.147095	-0.465152	0.280370
10	6	0	1.793560	0.666259	-0.073694
11	6	0	-3.234608	-0.431600	-0.124091
12	6	0	-4.710653	-0.167497	0.060775
13	6	0	3.560442	-0.649276	-0.073509
14	6	0	-5.557755	-1.295364	-0.489971
15	1	0	-0.995732	0.528447	-1.155477
16	1	0	-0.246767	1.916370	1.447024
17	1	0	-0.908358	2.755452	0.041577
18	1	0	-0.694698	-1.524500	0.059881
19	1	0	-0.535415	-0.678737	1.595568
20	1	0	1.655099	2.769948	0.204153
21	1	0	1.015942	2.196264	-1.325722
22	1	0	-2.820025	1.528421	0.048550
23	1	0	-2.969770	-1.363835	0.391460
24	1	0	-3.027675	-0.602862	-1.197672
25	1	0	-4.969590	0.776740	-0.437680

26	1	0	-4.921938	-0.022418	1.127748
27	1	0	-5.378905	-1.436410	-1.561593
28	1	0	-5.324246	-2.241959	0.009870
29	1	0	-6.625723	-1.103603	-0.354411
30	1	0	5.465484	-0.426225	-0.650744
31	1	0	5.032878	-2.026958	-0.319728

----

quinpirole

Input orientation:

Center Number	Atomic Number	Atomic Type	Coordinates (Angstroms) X	Y	Z
1	7	0	-1.781171	-0.152273	-0.281639
2	7	0	3.769924	0.046402	0.243037
3	7	0	4.048491	1.356551	0.072877
4	6	0	0.352348	-1.431575	-0.296668
5	6	0	-0.378079	-0.164230	0.171875
6	6	0	-0.404771	-2.691666	0.076247
7	6	0	1.766804	-1.515683	0.279791
8	6	0	0.354075	1.094662	-0.308150
9	6	0	-1.841383	-2.616238	-0.379336
10	6	0	-2.480137	-1.359250	0.157036
11	6	0	2.452432	-0.217591	0.133222
12	6	0	1.825477	0.975536	-0.122743
13	6	0	-2.470126	1.028814	0.243117
14	6	0	-3.932632	1.156237	-0.135040
15	6	0	2.864879	1.918923	-0.147968
16	6	0	-4.451697	2.536141	0.218613
17	1	0	0.427064	-1.371807	-1.395210
18	1	0	-0.361426	-0.172677	1.285398
19	1	0	-0.378874	-2.801190	1.171472
20	1	0	0.108336	-3.567040	-0.339734
21	1	0	2.320435	-2.322261	-0.214936
22	1	0	1.705450	-1.789734	1.343375
23	1	0	-0.007867	1.978235	0.225225
24	1	0	0.109453	1.252261	-1.368862
25	1	0	-1.891259	-2.610227	-1.476389
26	1	0	-2.410517	-3.486621	-0.035933
27	1	0	-3.516322	-1.305547	-0.183137
28	1	0	-2.507317	-1.401905	1.266161
29	1	0	-1.962735	1.921781	-0.132107
30	1	0	-2.380538	1.059153	1.349296
31	1	0	-4.050164	0.974034	-1.211153
32	1	0	-4.538300	0.402376	0.378982

33	1	0	4.516490	-0.609297	0.431209
34	1	0	2.802398	2.986356	-0.318335
35	1	0	-3.919794	3.314651	-0.338917
36	1	0	-5.517914	2.635584	-0.002715
37	1	0	-4.314333	2.744519	1.285838

----

aripiprazole

Standard orientation:

Center Number	Atomic Number	Atomic Type	Coordinates (Angstroms) X	Y	Z
1	17	0	6.454667	-1.844471	-0.800896
2	17	0	9.471924	-0.999884	-0.942102
3	8	0	-4.203172	-0.553247	0.484324
4	8	0	-11.029918	-1.673377	-0.223014
5	7	0	1.916153	-0.118744	1.078667
6	7	0	4.604104	0.312351	0.141580
7	7	0	-8.821687	-1.242740	-0.280412
8	6	0	2.944281	-1.151266	1.150260
9	6	0	2.227598	0.774358	-0.032553
10	6	0	4.321806	-0.562566	1.284047
11	6	0	3.606577	1.371916	0.101613
12	6	0	0.626543	-0.767730	0.877156
13	6	0	-0.559556	0.167853	0.803054
14	6	0	5.946452	0.704179	0.035663
15	6	0	-1.857329	-0.618306	0.783891
16	6	0	-3.035714	0.260353	0.466467
17	6	0	6.922952	-0.227035	-0.361297
18	6	0	6.364169	2.011636	0.278924
19	6	0	-7.920930	0.975282	-0.563759
20	6	0	-9.288924	1.403307	-0.992197
21	6	0	-7.725592	-0.367555	-0.233697
22	6	0	-5.388676	0.015326	0.140658
23	6	0	8.254691	0.150114	-0.459771
24	6	0	7.692460	2.378989	0.154851
25	6	0	-10.352620	0.629964	-0.232091
26	6	0	-6.478859	-0.849019	0.113775
27	6	0	8.650733	1.450890	-0.202367
28	6	0	-6.825299	1.815374	-0.513122
29	6	0	-5.557809	1.359560	-0.166003
30	6	0	-10.119651	-0.850013	-0.257109
31	1	0	2.738624	-1.797275	2.010007
32	1	0	2.916570	-1.791052	0.245794
33	1	0	2.151902	0.232589	-0.995767
34	1	0	1.502752	1.592098	-0.058327

35	1	0	4.400507	0.018791	2.219619
36	1	0	5.057860	-1.366674	1.334030
37	1	0	3.803414	2.019154	-0.757746
38	1	0	3.642208	1.997235	1.010964
39	1	0	0.652864	-1.380310	-0.046742
40	1	0	0.479548	-1.470143	1.708207
41	1	0	-0.550898	0.858823	1.656180
42	1	0	-0.503259	0.783111	-0.104070
43	1	0	-1.796421	-1.414812	0.030436
44	1	0	-2.016173	-1.108361	1.752779
45	1	0	-2.919553	0.716539	-0.526448
46	1	0	-3.134706	1.071499	1.201790
47	1	0	5.635311	2.754704	0.580748
48	1	0	-9.419403	2.478724	-0.845845
49	1	0	-9.411320	1.214406	-2.068260
50	1	0	-11.357651	0.821254	-0.612307
51	1	0	-10.344930	0.918229	0.828655
52	1	0	7.987413	3.403787	0.354304
53	1	0	-6.339835	-1.897077	0.365276
54	1	0	-6.959660	2.865490	-0.761476
55	1	0	9.696366	1.722516	-0.290867
56	1	0	-4.725739	2.052542	-0.141654
57	1	0	-8.646946	-2.238491	-0.185996

----

cariprazine

Standard orientation:

Center Number	Atomic Number	Atomic Type	Coordinates (Angstroms) X	Y	Z
1	17	0	-5.560267	-1.659357	-0.787834
2	17	0	-8.429533	-0.406141	-0.914201
3	8	0	7.952821	-0.411463	-1.265869
4	7	0	-0.607250	-0.124609	0.028679
5	7	0	6.445752	-0.057794	0.392631
6	7	0	-3.460425	0.152152	0.358258
7	7	0	7.285153	1.725822	-0.842249
8	6	0	2.993969	-1.246992	0.755942
9	6	0	3.958558	-0.982062	1.904915
10	6	0	3.673398	-0.915644	-0.568822
11	6	0	5.946304	-1.434464	0.399628
12	6	0	5.276110	-1.724175	1.733206
13	6	0	4.970669	-1.687431	-0.745229
14	6	0	1.696153	-0.473553	0.951234
15	6	0	0.575799	-0.977738	0.066870
16	6	0	-1.560098	-0.691465	-0.917136

17	6	0	-1.244651	-0.053840	1.338801
18	6	0	-2.836260	0.103233	-0.969985
19	6	0	-2.518777	0.753191	1.293860
20	6	0	7.261607	0.376997	-0.607966
21	6	0	-4.756999	0.692546	0.347441
22	6	0	-5.823911	-0.042500	-0.199353
23	6	0	-5.048387	1.943990	0.888363
24	6	0	8.431663	2.273095	-1.544443
25	6	0	6.623644	2.657857	0.053077
26	6	0	-7.103984	0.493026	-0.228319
27	6	0	-6.333750	2.456435	0.871763
28	6	0	-7.370008	1.742987	0.303043
29	1	0	2.760281	-2.326289	0.754926
30	1	0	4.128809	0.104732	1.974981
31	1	0	3.494112	-1.272555	2.854709
32	1	0	3.006639	-1.123161	-1.412781
33	1	0	3.877268	0.167522	-0.595892
34	1	0	6.814039	-2.090472	0.286699
35	1	0	5.080097	-2.803315	1.774308
36	1	0	5.963880	-1.493453	2.554279
37	1	0	5.445908	-1.434366	-1.699434
38	1	0	4.753510	-2.763943	-0.773448
39	1	0	1.399583	-0.545387	2.005742
40	1	0	1.882318	0.592366	0.750151
41	1	0	0.293432	-1.998999	0.394138
42	1	0	0.933336	-1.074494	-0.965043
43	1	0	-1.800366	-1.739054	-0.645584
44	1	0	-1.104447	-0.710904	-1.912668
45	1	0	-0.564922	0.419838	2.052213
46	1	0	-1.463570	-1.072412	1.715748
47	1	0	5.821558	0.624277	0.800368
48	1	0	-3.518133	-0.353462	-1.689093
49	1	0	-2.629637	1.132356	-1.313286
50	1	0	-2.273809	1.790968	1.007662
51	1	0	-2.967021	0.777838	2.291251
52	1	0	-4.250622	2.534263	1.323216
53	1	0	9.233427	2.548813	-0.847009
54	1	0	8.127599	3.169973	-2.088489
55	1	0	8.819707	1.548453	-2.256888
56	1	0	5.554897	2.442381	0.145964
57	1	0	7.075595	2.671429	1.053064
58	1	0	6.708066	3.659400	-0.370529
59	1	0	-6.526906	3.435742	1.297013
60	1	0	-8.379497	2.137122	0.272702

----

risperidone

Standard orientation:

Center Number	Atomic Number	Atomic Type	Coordinates (Angstroms) X	Y	Z
1	9	0	-9.057966	1.316077	1.359203
2	8	0	-6.118724	-0.855690	-1.679943
3	8	0	3.536463	1.049401	-1.499097
4	7	0	-0.097636	-1.073872	-0.054514
5	7	0	5.419546	0.740543	-0.258669
6	7	0	-4.707320	-0.893432	-1.723483
7	7	0	5.733080	-1.195201	1.008238
8	6	0	-2.818458	-0.048052	-0.421931
9	6	0	-1.904764	-0.370083	-1.590643
10	6	0	-2.420970	-0.916449	0.772068
11	6	0	-0.455711	-0.216465	-1.185669
12	6	0	-0.953276	-0.739903	1.083143
13	6	0	1.287759	-0.828532	0.331394
14	6	0	-4.266119	-0.202403	-0.714506
15	6	0	2.325690	-1.358672	-0.645438
16	6	0	3.696327	-0.932744	-0.221845
17	6	0	-5.348072	0.333642	0.052660
18	6	0	5.889135	2.041595	-0.780432
19	6	0	7.086592	2.576800	-0.040025
20	6	0	6.158955	-0.048171	0.564898
21	6	0	8.116346	1.485189	0.138909
22	6	0	7.510099	0.408450	1.010358
23	6	0	4.162151	0.325501	-0.717403
24	6	0	4.494648	-1.631867	0.638313
25	6	0	-6.478442	-0.114458	-0.612124
26	6	0	-5.470461	1.135251	1.186373
27	6	0	4.103087	-2.925831	1.258220
28	6	0	-7.770143	0.186482	-0.214523
29	6	0	-6.739098	1.456262	1.613948
30	6	0	-7.842738	0.975613	0.908028
31	1	0	-2.664031	1.003428	-0.131536
32	1	0	-2.114522	0.291494	-2.437791
33	1	0	-2.088591	-1.396957	-1.930764
34	1	0	-2.633645	-1.967305	0.532920
35	1	0	-3.015629	-0.655213	1.654030
36	1	0	0.185592	-0.457853	-2.037441
37	1	0	-0.257589	0.843459	-0.925227
38	1	0	-0.773324	0.308659	1.395674
39	1	0	-0.669287	-1.374873	1.928530
40	1	0	1.457517	-1.305033	1.305547
41	1	0	1.452838	0.257097	0.483629

42	1	0	2.254484	-2.448387	-0.704952
43	1	0	2.137393	-0.972247	-1.650487
44	1	0	5.045955	2.731098	-0.726475
45	1	0	6.122841	1.901737	-1.841614
46	1	0	6.782240	2.957296	0.943337
47	1	0	7.480686	3.424103	-0.607355
48	1	0	9.030164	1.867566	0.600179
49	1	0	8.393500	1.070415	-0.838936
50	1	0	7.391869	0.788896	2.034631
51	1	0	8.143138	-0.479005	1.084191
52	1	0	-4.595710	1.499444	1.715163
53	1	0	3.129329	-3.290457	0.933202
54	1	0	4.082799	-2.814740	2.347574
55	1	0	4.857396	-3.687158	1.035396
56	1	0	-8.656326	-0.158770	-0.733163
57	1	0	-6.908231	2.075728	2.487584

----

risperidoneH+

Standard orientation:

Center Number	Atomic Number	Atomic Type	Coordinates (Angstroms) X	Y	Z
1	7	0	-0.120126	0.692020	-0.041626
2	7	0	5.597775	-0.684113	0.227189
3	7	0	-4.716678	1.064552	1.595328
4	7	0	5.729729	1.345177	-0.921763
5	6	0	-2.905472	-0.079095	0.426430
6	6	0	-1.983657	0.354988	1.552836
7	6	0	-2.452269	0.588288	-0.872568
8	6	0	-0.546203	0.030898	1.234959
9	6	0	-1.010349	0.271444	-1.170934
10	6	0	1.310547	0.419118	-0.380809
11	6	0	-4.338115	0.221350	0.681417
12	6	0	2.278622	1.034676	0.606989
13	6	0	3.689356	0.768627	0.182042
14	6	0	-5.461627	-0.316018	-0.020855
15	6	0	6.197639	-1.951529	0.695570
16	6	0	7.479710	-2.291614	-0.016058
17	6	0	6.275185	0.238697	-0.503197
18	6	0	8.383965	-1.081259	-0.056022
19	6	0	7.700127	-0.012003	-0.877454
20	6	0	4.277642	-0.452593	0.635035
21	6	0	4.429825	1.604331	-0.605788
22	6	0	-6.547595	0.301866	0.578838

23	6	0	-5.656526	-1.237171	-1.048977
24	6	0	3.908561	2.876669	-1.172601
25	6	0	-7.861896	0.064185	0.215089
26	6	0	-6.950065	-1.500429	-1.439643
27	6	0	-8.006050	-0.847302	-0.802546
28	8	0	-6.122355	1.135897	1.550024
29	8	0	3.705824	-1.287564	1.342193
30	9	0	-9.247190	-1.136265	-1.216225
31	1	0	-2.820590	-1.168509	0.293642
32	1	0	-2.246197	-0.141847	2.490893
33	1	0	-2.082304	1.435065	1.720350
34	1	0	-2.583246	1.675126	-0.781898
35	1	0	-3.060026	0.256080	-1.719105
36	1	0	0.117655	0.377343	2.026857
37	1	0	-0.388085	-1.041894	1.081656
38	1	0	-0.847329	-0.803927	-1.296149
39	1	0	-0.659069	0.787793	-2.065721
40	1	0	1.473015	0.830795	-1.380944
41	1	0	1.415072	-0.669349	-0.432560
42	1	0	2.087774	2.109705	0.681739
43	1	0	2.132424	0.607538	1.602523
44	1	0	5.446162	-2.729862	0.557769
45	1	0	6.367024	-1.853055	1.773558
46	1	0	7.264781	-2.624541	-1.039470
47	1	0	7.941840	-3.132459	0.508058
48	1	0	9.356241	-1.319818	-0.493823
49	1	0	8.565051	-0.720312	0.964679
50	1	0	7.697067	-0.307105	-1.935906
51	1	0	8.217801	0.949393	-0.830943
52	1	0	-4.818336	-1.733754	-1.526786
53	1	0	2.834893	3.008362	-1.040953
54	1	0	4.137200	2.918035	-2.241541
55	1	0	4.422354	3.725132	-0.707259
56	1	0	-8.712804	0.543736	0.683583
57	1	0	-7.175005	-2.205522	-2.231955
58	1	0	-0.229418	1.707368	0.083594

----

clozapine

Input orientation:

Center Number	Atomic Number	Atomic Type	Coordinates (Angstroms) X	Y	Z
1	6	0	-5.549868	-1.573683	0.882633
2	6	0	-4.742215	-0.696897	1.587083
3	6	0	-3.976689	0.259900	0.937893

4	6	0	-4.014905	0.364279	-0.461666
5	6	0	-4.822357	-0.530020	-1.166523
6	6	0	-5.561818	-1.481268	-0.495692
7	1	0	-6.148494	-2.316859	1.397310
8	1	0	-4.698890	-0.754777	2.671727
9	1	0	-4.875197	-0.450105	-2.247581
10	7	0	-3.414247	1.395530	-1.173347
11	6	0	-1.318399	1.309118	0.113996
12	6	0	-1.787592	1.013680	1.394624
13	6	0	0.027917	1.094706	-0.177495
14	6	0	-0.901168	0.581672	2.374066
15	6	0	0.437907	0.407194	2.077428
16	6	0	0.903894	0.641041	0.790680
17	1	0	0.388193	1.306779	-1.180187
18	1	0	1.948754	0.480706	0.547226
19	1	0	1.119413	0.068566	2.851606
20	1	0	-1.280245	0.372850	3.371057
21	6	0	-2.236840	1.851314	-0.914318
22	7	0	-1.723019	2.851013	-1.705291
23	6	0	-0.965316	3.943123	-1.087199
24	6	0	-2.519902	3.334498	-2.823476
25	6	0	-1.641875	4.063486	-3.809859
26	6	0	-0.111982	4.644979	-2.108564
27	7	0	-0.921365	5.172387	-3.198113
28	6	0	-0.056343	5.781731	-4.191042
29	1	0	0.667216	5.057756	-4.606128
30	1	0	-0.654082	6.177577	-5.016744
31	1	0	-0.926025	3.346747	-4.257883
32	1	0	-2.263974	4.449428	-4.623824
33	1	0	-2.990928	2.484448	-3.317982
34	1	0	-3.324937	3.994276	-2.463958
35	1	0	0.652885	3.943546	-2.497093
36	1	0	0.420559	5.470511	-1.625702
37	1	0	-0.337190	3.563246	-0.282361
38	1	0	-1.673285	4.661353	-0.644150
39	17	0	-6.561035	-2.590971	-1.420895
40	7	0	-3.164953	1.152974	1.682687
41	1	0	0.506990	6.607072	-3.747182
42	1	0	-3.327168	1.019947	2.677741

----

clozapineH+

Input orientation:

Center Number	Atomic Number	Atomic Type	Coordinates (Angstroms) X	Y	Z

1	7	0	-3.396143	1.391087	-1.168361
2	7	0	-1.690736	2.820660	-1.719560
3	7	0	-0.900271	5.134150	-3.221941
4	7	0	-3.149874	1.130580	1.687131
5	6	0	-5.577587	-1.551926	0.873001
6	6	0	-4.754585	-0.694003	1.582063
7	6	0	-3.972622	0.253279	0.937657
8	6	0	-4.013961	0.365349	-0.459999
9	6	0	-4.837436	-0.508327	-1.170461
10	6	0	-5.591581	-1.451565	-0.504830
11	6	0	-1.298729	1.292844	0.119384
12	6	0	-1.773245	0.996404	1.398929
13	6	0	0.052771	1.094443	-0.161957
14	6	0	-0.887507	0.574335	2.383431
15	6	0	0.454838	0.413062	2.095657
16	6	0	0.927113	0.651541	0.812240
17	6	0	-2.215366	1.826435	-0.912556
18	6	0	-0.969955	3.925306	-1.088190
19	6	0	-2.507322	3.298589	-2.823204
20	6	0	-1.655902	4.005510	-3.843663
21	6	0	-0.084488	4.623269	-2.080693
22	6	0	-0.065155	5.842972	-4.217963
23	17	0	-6.612972	-2.536567	-1.433493
24	1	0	-6.188881	-2.287195	1.384218
25	1	0	-4.712845	-0.758673	2.666284
26	1	0	-4.891233	-0.417147	-2.250597
27	1	0	0.419860	1.308422	-1.161577
28	1	0	1.975125	0.502388	0.575614
29	1	0	1.134385	0.082199	2.874839
30	1	0	-1.270528	0.363865	3.378478
31	1	0	0.663700	5.135076	-4.614244
32	1	0	-0.708460	6.209496	-5.017267
33	1	0	-0.905159	3.339709	-4.278713
34	1	0	-2.272263	4.423045	-4.641398
35	1	0	-2.994951	2.455686	-3.311473
36	1	0	-3.298934	3.972212	-2.458815
37	1	0	0.661155	3.946266	-2.509403
38	1	0	0.419644	5.477287	-1.625740
39	1	0	-0.352288	3.569197	-0.265065
40	1	0	-1.692577	4.645220	-0.671396
41	1	0	0.442379	6.671424	-3.725004
42	1	0	-3.312201	0.993621	2.681541
43	1	0	-1.587214	5.797476	-2.841903

----

olanzapine

Input orientation:

Center Number	Atomic Number	Atomic Type	Coordinates (Angstroms) X	Y	Z
1	6	0	-5.796989	-1.631992	0.742911
2	6	0	-5.052040	-0.790489	1.552584
3	6	0	-4.248768	0.197545	1.003388
4	6	0	-4.174793	0.368145	-0.387858
5	6	0	-4.927395	-0.500161	-1.181881
6	6	0	-5.722088	-1.491780	-0.634563
7	1	0	-6.428876	-2.393427	1.188696
8	1	0	-5.089062	-0.889678	2.634875
9	1	0	-4.887545	-0.362217	-2.258776
10	7	0	-3.510100	1.412396	-1.035411
11	6	0	-1.546189	1.352786	0.422023
12	6	0	-2.140179	1.041979	1.610140
13	6	0	-0.129591	1.162523	0.462069
14	6	0	0.340307	0.734511	1.656422
15	1	0	0.526908	1.351837	-0.382668
16	6	0	-2.334588	1.836550	-0.716784
17	7	0	-1.715781	2.761504	-1.529695
18	6	0	-0.986080	3.874993	-0.918407
19	6	0	-2.406620	3.192311	-2.736437
20	6	0	-1.432627	3.833172	-3.692713
21	6	0	-0.031252	4.488487	-1.907070
22	7	0	-0.731527	4.961345	-3.093639
23	6	0	0.230228	5.484572	-4.044639
24	1	0	0.971159	4.720841	-4.341056
25	1	0	-0.281907	5.829610	-4.946965
26	1	0	-0.705770	3.071450	-4.036338
27	1	0	-1.976441	4.182609	-4.576122
28	1	0	-2.863679	2.324064	-3.212072
29	1	0	-3.216766	3.895109	-2.485403
30	1	0	0.743316	3.746520	-2.185439
31	1	0	0.483509	5.331834	-1.435728
32	1	0	-0.438537	3.533133	-0.040565
33	1	0	-1.707536	4.636888	-0.582562
34	7	0	-3.511462	1.066534	1.855999
35	1	0	0.769650	6.331078	-3.610596
36	1	0	-3.748799	0.918406	2.832144
37	1	0	-6.294611	-2.145082	-1.285461
38	6	0	1.741270	0.476876	2.070040
39	1	0	2.410757	0.669719	1.227488
40	1	0	2.048449	1.123478	2.898630
41	1	0	1.892889	-0.558213	2.392378

42	16	0	-0.975382	0.515495	2.787879

----

olanzapineH+

Input orientation:

Center Number	Atomic Number	Atomic Type	Coordinates (Angstroms) X	Y	Z
1	7	0	-0.413412	-1.598780	-0.064111
2	7	0	1.647430	-0.582140	-0.024811
3	7	0	4.482518	-0.868099	-0.323856
4	7	0	-2.250828	0.279616	-1.321639
5	6	0	-4.567388	-2.133290	0.274944
6	6	0	-4.085878	-1.055339	-0.449143
7	6	0	-2.723209	-0.818728	-0.549722
8	6	0	-1.802991	-1.669871	0.080078
9	6	0	-2.313580	-2.744655	0.810005
10	6	0	-3.673452	-2.975499	0.918306
11	6	0	-0.298316	0.844076	0.046170
12	6	0	-1.458829	1.165949	-0.596488
13	6	0	0.254310	1.958999	0.751145
14	6	0	-0.463213	3.099842	0.635997
15	6	0	0.263650	-0.505842	-0.040235
16	6	0	2.407787	0.318879	-0.886973
17	6	0	2.260229	-1.898760	0.016573
18	6	0	3.674940	-1.800031	0.521539
19	6	0	3.816979	0.468753	-0.388556
20	6	0	5.874780	-0.760138	0.167707
21	6	0	-0.176634	4.442439	1.197219
22	16	0	-1.888052	2.829755	-0.341980
23	1	0	-5.636335	-2.310633	0.335401
24	1	0	-4.770530	-0.378269	-0.954382
25	1	0	-1.603909	-3.417429	1.283191
26	1	0	1.174117	1.914606	1.327459
27	1	0	5.846543	-0.351756	1.178492
28	1	0	6.322376	-1.753375	0.173492
29	1	0	3.714062	-1.399416	1.538302
30	1	0	4.167829	-2.773125	0.498650
31	1	0	1.699545	-2.543831	0.692157
32	1	0	2.245135	-2.370336	-0.978672
33	1	0	3.849723	0.880960	0.624653
34	1	0	4.407671	1.098073	-1.056037
35	1	0	1.945635	1.304841	-0.923659
36	1	0	2.422437	-0.077570	-1.915181
37	1	0	6.430070	-0.094974	-0.492767
38	1	0	-3.007298	0.732438	-1.825407

39	1	0	-4.032920	-3.823148	1.493035
40	1	0	0.750388	4.404478	1.775482
41	1	0	-0.054566	5.195803	0.412111
42	1	0	-0.975052	4.789870	1.860656
43	1	0	4.503238	-1.251343	-1.277148

quetiapine

Standard orientation:

Center Number	Atomic Number	Atomic Type	Coordinates (Angstroms) X	Y	Z
1	6	0	-6.270470	-0.798992	0.286347
2	6	0	-5.513450	-0.031591	-0.581597
3	6	0	-4.132436	-0.174439	-0.631372
4	6	0	-3.475221	-1.101489	0.194685
5	6	0	-4.261825	-1.861823	1.067034
6	6	0	-5.633525	-1.708875	1.120084
7	1	0	-7.348843	-0.682749	0.316028
8	1	0	-5.992359	0.696322	-1.229973
9	1	0	-3.764012	-2.589459	1.701679
10	1	0	-6.213582	-2.311418	1.812493
11	7	0	-2.123582	-1.397266	0.110864
12	16	0	-3.191574	0.831348	-1.767940
13	6	0	-1.374311	0.938287	0.302484
14	6	0	-2.236957	1.670836	-0.512631
15	6	0	-0.704885	1.598009	1.331486
16	6	0	-2.384936	3.039520	-0.332374
17	6	0	-1.687315	3.685874	0.673433
18	6	0	-0.860695	2.959688	1.518011
19	1	0	-0.047606	1.033892	1.986398
20	1	0	-0.330321	3.457463	2.323139
21	1	0	-1.803280	4.756490	0.808341
22	1	0	-3.057680	3.593508	-0.979709
23	6	0	-1.178776	-0.522160	0.110058
24	7	0	0.123630	-0.946056	0.057306
25	6	0	1.129101	-0.155179	-0.651574
26	6	0	0.393886	-2.371770	-0.057117
27	6	0	1.798797	-2.676909	0.396218
28	6	0	2.516107	-0.493565	-0.171872
29	7	0	2.800987	-1.914224	-0.336690
30	6	0	4.117358	-2.267985	0.188720
31	6	0	5.271415	-1.533952	-0.440442
32	8	0	5.476245	-0.299163	0.221852
33	6	0	6.254069	0.604698	-0.531863
34	6	0	6.259083	1.917415	0.190922

35	8	0	4.957074	2.473840	0.284285
36	1	0	4.384647	1.806579	0.684693
37	1	0	6.694058	1.789398	1.191911
38	1	0	6.878171	2.633595	-0.355236
39	1	0	7.283039	0.232161	-0.653459
40	1	0	5.813060	0.726349	-1.534848
41	1	0	6.185576	-2.140782	-0.368757
42	1	0	5.083552	-1.355818	-1.509972
43	1	0	4.159524	-2.103943	1.282667
44	1	0	4.253823	-3.342499	0.027326
45	1	0	1.883001	-2.475781	1.481749
46	1	0	1.992560	-3.745058	0.252595
47	1	0	-0.314220	-2.918930	0.565362
48	1	0	0.246115	-2.702661	-1.097078
49	1	0	2.624243	-0.191960	0.889436
50	1	0	3.233771	0.093684	-0.752211
51	1	0	0.948482	0.909529	-0.505383
52	1	0	1.049579	-0.357647	-1.731450

----

quetiapineH+

Standard orientation:

Center Number	Atomic Number	Atomic Type	Coordinates (Angstroms) X	Y	Z
1	7	0	2.133276	-1.380406	-0.118791
2	7	0	-0.112791	-0.925435	-0.108900
3	7	0	-2.794203	-1.887898	0.251286
4	6	0	6.288434	-0.833310	-0.233395
5	6	0	5.526998	-0.045196	0.611623
6	6	0	4.143658	-0.169858	0.639095
7	6	0	3.491231	-1.100279	-0.185219
8	6	0	4.280808	-1.881587	-1.034585
9	6	0	5.654932	-1.745508	-1.066834
10	6	0	1.389419	0.958334	-0.327698
11	6	0	2.253085	1.689496	0.488326
12	6	0	0.705304	1.620644	-1.345375
13	6	0	2.395293	3.058998	0.312414
14	6	0	1.689488	3.707085	-0.686766
15	6	0	0.856039	2.983561	-1.526716
16	6	0	1.198911	-0.500934	-0.139771
17	6	0	-1.104273	-0.146875	0.626472
18	6	0	-0.370720	-2.349839	0.013927
19	6	0	-1.754256	-2.678129	-0.476914
20	6	0	-2.495318	-0.427018	0.127121
21	6	0	-4.160919	-2.281903	-0.216619

22	6	0	-5.263388	-1.526206	0.464741
23	6	0	-6.217187	0.620566	0.574427
24	6	0	-6.236664	1.925930	-0.160572
25	8	0	-5.458184	-0.294183	-0.191160
26	8	0	-4.940455	2.491193	-0.273004
27	16	0	3.199297	0.855856	1.753305
28	1	0	7.368614	-0.731946	-0.244841
29	1	0	6.004187	0.683898	1.259838
30	1	0	3.785002	-2.611959	-1.667462
31	1	0	6.239062	-2.364360	-1.740993
32	1	0	0.039061	1.057199	-1.992465
33	1	0	0.316125	3.484290	-2.323497
34	1	0	1.801685	4.778572	-0.817541
35	1	0	3.068153	3.613101	0.959465
36	1	0	-4.376351	1.845513	-0.717324
37	1	0	-6.683599	1.785763	-1.154443
38	1	0	-6.853946	2.641726	0.388080
39	1	0	-7.242096	0.247978	0.720628
40	1	0	-5.751071	0.747224	1.564831
41	1	0	-6.182162	-2.127599	0.430870
42	1	0	-5.020694	-1.364775	1.527522
43	1	0	-4.185831	-2.119605	-1.297838
44	1	0	-4.247431	-3.351905	-0.019065
45	1	0	-1.873406	-2.433080	-1.535946
46	1	0	-1.979338	-3.735036	-0.325168
47	1	0	0.345148	-2.906062	-0.589908
48	1	0	-0.247821	-2.675692	1.058533
49	1	0	-2.615309	-0.169173	-0.930028
50	1	0	-3.227139	0.123013	0.720245
51	1	0	-0.915945	0.921315	0.526317
52	1	0	-1.044253	-0.392910	1.699041
53	1	0	-2.729501	-2.126917	1.250319

----

ziprasidone

Input orientation:

Center Number	Atomic Number	Atomic Type	Coordinates (Angstroms) X	Y	Z
1	17	0	-3.773471	-3.250371	0.957244
2	16	0	6.761759	-1.327203	-0.759429
3	8	0	-8.234400	2.287328	-1.094827
4	7	0	0.292682	-1.245058	0.097344
5	7	0	3.044830	-0.554184	-0.455391
6	7	0	5.108853	-1.622314	-0.797433
7	7	0	-7.263437	0.289646	-0.516207

8	6	0	0.941641	-0.091079	0.712417
9	6	0	0.934464	-1.502713	-1.189390
10	6	0	2.426813	-0.293871	0.852509
11	6	0	2.417956	-1.725777	-1.057252
12	6	0	-1.116116	-0.970929	-0.148681
13	6	0	-1.948289	-0.745663	1.102546
14	6	0	4.433009	-0.562831	-0.442875
15	6	0	-3.386819	-0.558676	0.728962
16	6	0	5.219045	0.601940	-0.102613
17	6	0	-5.145639	0.928458	0.011722
18	6	0	-3.849230	0.725911	0.418214
19	6	0	-6.011674	-0.158419	-0.089937
20	6	0	-4.289313	-1.607792	0.602926
21	6	0	-5.894942	2.157399	-0.380250
22	6	0	6.583489	0.311720	-0.216017
23	6	0	-5.610766	-1.440904	0.203600
24	6	0	-7.273518	1.637731	-0.715248
25	6	0	4.823886	1.903527	0.225507
26	6	0	7.562277	1.269951	0.037118
27	6	0	5.787672	2.855365	0.470701
28	6	0	7.149217	2.535704	0.388587
29	1	0	0.527241	0.074013	1.710689
30	1	0	0.746589	0.823435	0.118580
31	1	0	0.752407	-0.659650	-1.882619
32	1	0	0.479418	-2.390873	-1.639407
33	1	0	2.869576	0.591521	1.310494
34	1	0	2.628156	-1.145343	1.523716
35	1	0	2.616162	-2.627761	-0.456292
36	1	0	2.849207	-1.890626	-2.047454
37	1	0	-1.222259	-0.084723	-0.806330
38	1	0	-1.528579	-1.821840	-0.705516
39	1	0	-1.609602	0.155043	1.624767
40	1	0	-1.821750	-1.589168	1.789062
41	1	0	-3.158155	1.561620	0.503798
42	1	0	-5.972545	2.902405	0.418146
43	1	0	-5.477994	2.669089	-1.253646
44	1	0	-6.285540	-2.286077	0.124434
45	1	0	-8.068754	-0.302446	-0.679732
46	1	0	3.772455	2.168744	0.271152
47	1	0	8.616907	1.029478	-0.048061
48	1	0	5.492260	3.868325	0.723474
49	1	0	7.890620	3.302435	0.590196

----

ziprasidoneH+

Input orientation:

Center Number	Atomic Number	Atomic Type	Coordinates (Angstroms) X	Y	Z
1	7	0	0.287630	-0.939890	-0.068932
2	7	0	3.108701	-0.509731	-0.485656
3	7	0	5.089324	-1.754730	-0.669594
4	7	0	-7.448476	-0.074716	-0.323234
5	6	0	1.011022	0.214663	0.547386
6	6	0	0.989285	-1.362387	-1.320576
7	6	0	2.454103	-0.137407	0.772905
8	6	0	2.434469	-1.673886	-1.047446
9	6	0	-1.152550	-0.652730	-0.346295
10	6	0	-1.944055	-0.354118	0.911614
11	6	0	4.494732	-0.629065	-0.388996
12	6	0	-3.411443	-0.358582	0.607802
13	6	0	5.358479	0.473053	-0.031993
14	6	0	-5.408488	0.860903	0.038060
15	6	0	-4.068472	0.847173	0.341805
16	6	0	-6.117046	-0.338188	-0.003800
17	6	0	-4.161382	-1.527653	0.543523
18	6	0	-6.354196	1.970247	-0.277853
19	6	0	6.694520	0.055105	-0.052113
20	6	0	-5.517302	-1.551525	0.245473
21	6	0	-7.668325	1.259266	-0.502198
22	6	0	5.069689	1.814510	0.246619
23	6	0	7.742392	0.924585	0.239767
24	6	0	6.102606	2.677665	0.532637
25	6	0	7.430629	2.231807	0.539564
26	8	0	-8.740852	1.763575	-0.790584
27	17	0	-3.388010	-3.073969	0.852008
28	16	0	6.754620	-1.609579	-0.537078
29	1	0	0.533072	0.449859	1.498273
30	1	0	0.893963	1.056231	-0.141679
31	1	0	0.876757	-0.536147	-2.027984
32	1	0	0.469560	-2.240582	-1.706828
33	1	0	2.945629	0.720387	1.229181
34	1	0	2.521017	-0.974512	1.488276
35	1	0	2.507920	-2.538920	-0.368508
36	1	0	2.906107	-1.954506	-1.990440
37	1	0	-1.176115	0.187626	-1.046991
38	1	0	-1.541239	-1.537990	-0.857373
39	1	0	-1.670365	0.627542	1.307669
40	1	0	-1.703847	-1.098279	1.679870
41	1	0	-3.498907	1.772381	0.383937
42	1	0	-6.468951	2.697174	0.532718
43	1	0	-6.089226	2.534668	-1.177776
44	1	0	-6.069308	-2.484071	0.212379

45	1	0	-8.173301	-0.775347	-0.419889
46	1	0	4.048283	2.180737	0.223827
47	1	0	8.772756	0.585025	0.223945
48	1	0	5.888262	3.718807	0.750076
49	1	0	8.227316	2.931414	0.771880
50	1	0	0.331016	-1.724895	0.595621

haloperidol

Input orientation:

Center Number	Atomic Number	Atomic Type	Coordinates (Angstroms) X	Y	Z
1	17	0	9.164710	-0.149114	-0.516083
2	9	0	-8.966232	0.967812	-0.580835
3	8	0	2.880874	1.720565	-0.675012
4	8	0	-2.913037	-0.431086	-1.293629
5	7	0	0.591961	-0.582044	0.954572
6	6	0	3.164240	0.444271	-0.081790
7	6	0	2.347325	-0.640631	-0.771301
8	6	0	2.788804	0.449535	1.401182
9	6	0	0.867727	-0.510210	-0.480223
10	6	0	1.292505	0.513712	1.618393
11	6	0	-0.840316	-0.442692	1.194565
12	6	0	4.662250	0.264670	-0.228871
13	6	0	-1.652833	-1.675806	0.849071
14	6	0	5.241252	-0.902620	-0.710667
15	6	0	5.505234	1.302853	0.166654
16	6	0	-3.138665	-1.385453	0.873251
17	6	0	6.619469	-1.038219	-0.800590
18	6	0	6.880683	1.187160	0.082392
19	6	0	-3.642545	-0.654407	-0.340245
20	6	0	7.422051	0.010315	-0.402912
21	6	0	-5.064821	-0.230176	-0.371249
22	6	0	-5.936780	-0.476301	0.689135
23	6	0	-5.543524	0.430307	-1.503179
24	6	0	-7.258120	-0.073910	0.624899
25	6	0	-6.858844	0.839370	-1.584021
26	6	0	-7.687828	0.575383	-0.512441
27	1	0	2.678436	-1.623341	-0.417322
28	1	0	2.522304	-0.601320	-1.852238
29	1	0	3.273864	1.297668	1.896905
30	1	0	3.185153	-0.467836	1.854709
31	1	0	0.333479	-1.312298	-0.995165
32	1	0	0.468082	0.439065	-0.891978
33	1	0	1.076967	0.469904	2.690642

34	1	0	0.894408	1.488210	1.267014
35	1	0	-0.984156	-0.223747	2.259779
36	1	0	-1.228086	0.437224	0.644966
37	1	0	-1.428017	-2.464929	1.574388
38	1	0	-1.376833	-2.067047	-0.135022
39	1	0	1.968856	1.968418	-0.480177
40	1	0	4.625751	-1.735914	-1.030335
41	1	0	5.081160	2.227239	0.546475
42	1	0	-3.404902	-0.807313	1.768259
43	1	0	-3.720818	-2.313398	0.947465
44	1	0	7.059558	-1.953868	-1.181024
45	1	0	7.526344	2.002560	0.391035
46	1	0	-5.590688	-0.988607	1.580046
47	1	0	-4.867008	0.621284	-2.329228
48	1	0	-7.951563	-0.255467	1.438609
49	1	0	-7.248532	1.353931	-2.455573

----

trifluoperidol

Standard orientation:

Center Number	Atomic Number	Atomic Type	Coordinates (Angstroms) X	Y	Z
1	9	0	9.756775	-1.517833	-0.295430
2	8	0	-2.110119	-1.166960	-0.782396
3	8	0	3.854499	0.354342	-1.158075
4	7	0	0.327484	0.914725	0.923293
5	6	0	-2.281031	0.150257	-0.238047
6	6	0	-1.312641	1.119427	-0.903222
7	6	0	-1.991206	0.148190	1.263834
8	6	0	0.123371	0.833927	-0.522434
9	6	0	-0.526189	-0.077562	1.569107
10	6	0	1.722239	0.631899	1.245368
11	6	0	-3.739983	0.488096	-0.478236
12	6	0	2.669709	1.771354	0.921935
13	6	0	-4.160038	1.668065	-1.083093
14	6	0	-4.709634	-0.411222	-0.049608
15	6	0	4.117768	1.335442	0.993621
16	6	0	-5.508555	1.943741	-1.252799
17	6	0	-6.056152	-0.132386	-0.221535
18	6	0	4.581860	0.539487	-0.195164
19	6	0	-6.468657	1.046343	-0.822203
20	6	0	5.963386	-0.003467	-0.186571
21	6	0	6.852230	0.244336	0.859111
22	6	0	6.386240	-0.777875	-1.267475
23	6	0	8.137760	-0.263841	0.829013

24	6	0	7.664206	-1.296183	-1.312133
25	6	0	8.512904	-1.023307	-0.258442
26	1	0	-1.555569	2.141671	-0.592109
27	1	0	-1.427549	1.066316	-1.991650
28	1	0	-2.594141	-0.624832	1.752266
29	1	0	-2.304783	1.117306	1.672249
30	1	0	0.776339	1.553686	-1.022928
31	1	0	0.432076	-0.165767	-0.890435
32	1	0	-0.368235	-0.027341	2.651089
33	1	0	-0.222074	-1.100090	1.262738
34	1	0	1.786636	0.422179	2.320245
35	1	0	2.046647	-0.293968	0.731850
36	1	0	2.503118	2.585066	1.635608
37	1	0	2.462433	2.180343	-0.072166
38	1	0	-1.234441	-1.496397	-0.544601
39	1	0	-3.434841	2.392794	-1.436035
40	1	0	-4.413212	-1.343326	0.424552
41	1	0	4.301792	0.743888	1.901124
42	1	0	4.786533	2.201768	1.077715
43	1	0	-5.811904	2.870326	-1.728789
44	1	0	6.548885	0.844254	1.709814
45	1	0	5.695983	-0.971911	-2.081405
46	1	0	8.845227	-0.079442	1.629803
47	1	0	8.010694	-1.900907	-2.143035
48	1	0	-7.523398	1.260379	-0.953282
49	6	0	-7.043203	-1.142117	0.259191
50	9	0	-8.307850	-0.751095	0.102875
51	9	0	-6.917271	-2.312915	-0.386368
52	9	0	-6.882760	-1.421907	1.561899

----

benperidol

Standard orientation:

Center Number	Atomic Number	Atomic Type	Coordinates (Angstroms) X	Y	Z
1	9	0	-7.686653	3.051817	0.140236
2	8	0	2.414725	2.369229	0.591879
3	8	0	-4.648610	-2.248315	-1.215959
4	7	0	-0.011849	-1.580746	0.746990
5	7	0	3.674656	0.471581	0.073728
6	7	0	4.680059	2.430765	0.098663
7	6	0	2.657106	-0.569142	0.206065
8	6	0	1.521809	-0.410126	-0.788023
9	6	0	2.113020	-0.675771	1.618963
10	6	0	0.516855	-1.528650	-0.612501

11	6	0	1.087412	-1.785727	1.685203
12	6	0	-0.950308	-2.689678	0.892919
13	6	0	5.013123	0.255625	-0.238467
14	6	0	-2.187109	-2.619907	0.021133
15	6	0	3.467661	1.816464	0.289627
16	6	0	5.649097	1.500744	-0.225259
17	6	0	-2.985768	-1.352471	0.225694
18	6	0	5.713058	-0.898398	-0.530241
19	6	0	6.993776	1.628620	-0.502136
20	6	0	7.069824	-0.773045	-0.809296
21	6	0	-4.301218	-1.327102	-0.492830
22	6	0	7.699331	0.467042	-0.796001
23	6	0	-5.180706	-0.144922	-0.309751
24	6	0	-4.805570	0.940657	0.481341
25	6	0	-6.421937	-0.122460	-0.946649
26	6	0	-5.647510	2.026009	0.637661
27	6	0	-7.275521	0.951832	-0.800379
28	6	0	-6.865231	2.004622	-0.007721
29	1	0	3.172707	-1.510327	-0.023907
30	1	0	1.912495	-0.427960	-1.810827
31	1	0	1.029860	0.557501	-0.637191
32	1	0	1.655906	0.275245	1.914889
33	1	0	2.927657	-0.886759	2.319919
34	1	0	-0.306828	-1.382382	-1.316509
35	1	0	0.992246	-2.498268	-0.868975
36	1	0	1.585694	-2.754786	1.475590
37	1	0	0.674131	-1.856410	2.696731
38	1	0	-0.430705	-3.647766	0.693832
39	1	0	-1.260463	-2.716364	1.945509
40	1	0	-1.927809	-2.735010	-1.037327
41	1	0	-2.807313	-3.488257	0.270653
42	1	0	4.805995	3.428704	0.195560
43	1	0	-2.418584	-0.471411	-0.103515
44	1	0	-3.174019	-1.185294	1.295657
45	1	0	5.229073	-1.869299	-0.544345
46	1	0	7.477938	2.599453	-0.490128
47	1	0	7.645740	-1.662689	-1.042911
48	1	0	8.759379	0.531827	-1.019208
49	1	0	-3.845568	0.950306	0.985546
50	1	0	-6.715367	-0.965960	-1.562342
51	1	0	-5.373511	2.879689	1.247577
52	1	0	-8.244904	0.986052	-1.285545

----

droperidol

Input orientation:

Center Number	Atomic Number	Atomic Type	Coordinates (Angstroms) X	Y	Z
1	9	0	-9.663263	-0.276682	0.962632
2	8	0	4.319983	2.847391	-0.544862
3	8	0	-3.928785	1.432285	-0.861465
4	7	0	0.076026	0.210623	-0.217155
5	7	0	4.275761	0.558530	-0.105478
6	7	0	6.228336	1.574316	-0.186440
7	6	0	0.697206	0.599316	1.045200
8	6	0	2.055162	1.224485	0.816804
9	6	0	0.852450	-0.871189	-0.811438
10	6	0	2.865569	0.364258	-0.096792
11	6	0	-1.289797	-0.230524	0.041267
12	6	0	2.313568	-0.597963	-0.824295
13	6	0	-2.064240	-0.633813	-1.196782
14	6	0	5.255603	-0.410694	0.117626
15	6	0	-3.517464	-0.911750	-0.870648
16	6	0	4.880557	1.786290	-0.300571
17	6	0	6.490833	0.239401	0.064216
18	6	0	5.170419	-1.762608	0.380661
19	6	0	-4.366348	0.310490	-0.660635
20	6	0	7.673879	-0.441261	0.258842
21	6	0	6.361885	-2.451982	0.579992
22	6	0	7.591563	-1.804852	0.516166
23	6	0	-5.773105	0.122076	-0.225026
24	6	0	-6.326772	-1.143617	-0.033576
25	6	0	-6.566442	1.248555	-0.005921
26	6	0	-7.642663	-1.286375	0.366127
27	6	0	-7.880655	1.124756	0.395919
28	6	0	-8.389076	-0.146253	0.572450
29	1	0	0.038726	1.307985	1.555734
30	1	0	0.798265	-0.283332	1.706516
31	1	0	1.944106	2.232128	0.399898
32	1	0	2.580763	1.339667	1.772114
33	1	0	0.516950	-1.041355	-1.839866
34	1	0	0.669543	-1.824105	-0.271170
35	1	0	-1.284320	-1.085285	0.748671
36	1	0	-1.810449	0.589960	0.548767
37	1	0	2.927682	-1.232633	-1.457495
38	1	0	-1.990474	0.149832	-1.959789
39	1	0	-1.633821	-1.542032	-1.630355
40	1	0	-3.592065	-1.547255	0.021830
41	1	0	-3.994896	-1.490364	-1.672372
42	1	0	6.909018	2.310659	-0.312160
43	1	0	4.211559	-2.267689	0.428986
44	1	0	8.628529	0.072226	0.215233

45	1	0	6.327649	-3.516761	0.786603
46	1	0	8.503699	-2.371808	0.671797
47	1	0	-5.734354	-2.036479	-0.199283
48	1	0	-6.138791	2.234261	-0.153905
49	1	0	-8.091133	-2.261789	0.518867
50	1	0	-8.510574	1.989456	0.573865

----

pipamperone

Standard orientation:

Center Number	Atomic Number	Atomic Type	Coordinates (Angstroms) X	Y	Z
1	9	0	-8.988694	0.948744	0.332330
2	8	0	2.214376	2.400515	0.029251
3	8	0	-2.960655	0.385420	-1.249035
4	7	0	4.674573	0.093503	0.158963
5	7	0	0.566017	-1.192393	0.450000
6	7	0	3.505868	2.104476	1.835420
7	6	0	3.204680	0.191744	0.338895
8	6	0	2.732488	-0.702857	1.489489
9	6	0	2.400453	-0.206095	-0.903200
10	6	0	1.228659	-0.679580	1.645860
11	6	0	0.904110	-0.317069	-0.672910
12	6	0	5.197664	-1.258219	-0.052109
13	6	0	5.156810	0.967015	-0.914841
14	6	0	6.714656	-1.262670	-0.043381
15	6	0	6.669325	1.050370	-0.927651
16	6	0	2.926848	1.666017	0.709648
17	6	0	7.273478	-0.329835	-1.099751
18	6	0	-0.875801	-1.185716	0.669411
19	6	0	-1.669081	-1.932322	-0.384604
20	6	0	-3.159480	-1.741210	-0.198575
21	6	0	-3.676535	-0.404891	-0.654445
22	6	0	-5.096163	-0.070538	-0.378961
23	6	0	-5.979816	-0.998461	0.171130
24	6	0	-5.559481	1.209803	-0.683675
25	6	0	-7.299443	-0.662827	0.409995
26	6	0	-6.871456	1.564652	-0.443986
27	6	0	-7.713370	0.614570	0.097922
28	1	0	3.021963	-1.735997	1.277252
29	1	0	3.216818	-0.423721	2.430576
30	1	0	2.569721	0.500748	-1.720339
31	1	0	2.775119	-1.180729	-1.239208
32	1	0	0.943397	-1.305389	2.497547
33	1	0	0.871446	0.345064	1.875941

34	1	0	0.468065	0.683482	-0.501937
35	1	0	0.443985	-0.706744	-1.585768
36	1	0	4.839992	-1.921198	0.737073
37	1	0	4.849993	-1.677672	-1.014980
38	1	0	4.817692	0.602149	-1.902473
39	1	0	4.733669	1.968568	-0.785880
40	1	0	7.069332	-0.952200	0.948810
41	1	0	7.062947	-2.289235	-0.201740
42	1	0	6.982108	1.720907	-1.735436
43	1	0	7.016918	1.495063	0.014833
44	1	0	7.011283	-0.714631	-2.095925
45	1	0	8.366747	-0.291026	-1.050577
46	1	0	-1.070325	-1.653540	1.642892
47	1	0	-1.239063	-0.142493	0.750156
48	1	0	-1.433836	-3.000032	-0.317240
49	1	0	-1.385683	-1.611739	-1.391611
50	1	0	3.410873	3.080701	2.080377
51	1	0	4.182672	1.542224	2.329780
52	1	0	-3.441331	-1.884491	0.853126
53	1	0	-3.723616	-2.503023	-0.752905
54	1	0	-5.643819	-2.000566	0.413928
55	1	0	-4.873638	1.934408	-1.109136
56	1	0	-8.002775	-1.371938	0.832232
57	1	0	-7.248649	2.556504	-0.668304

----

spiperone

Standard orientation:

Center Number	Atomic Number	Atomic Type	Coordinates (Angstroms) X	Y	Z
1	9	0	-4.947289	2.707378	-0.404318
2	8	0	4.538020	-1.595904	-0.205760
3	8	0	-2.656067	-2.854255	1.316400
4	7	0	2.663627	1.371862	-0.308905
5	7	0	0.782688	-2.324114	0.262133
6	7	0	4.796839	0.669469	-0.115549
7	6	0	2.606693	-0.094615	-0.131942
8	6	0	1.889670	-0.792821	-1.292328
9	6	0	2.062893	-0.497336	1.250764
10	6	0	1.511352	-2.230421	-0.996214
11	6	0	1.680189	-1.958936	1.348465
12	6	0	4.083635	-0.459446	-0.161678
13	6	0	3.994661	1.851831	0.023153
14	6	0	0.236138	-3.654364	0.471132
15	6	0	1.600352	2.240759	-0.074763

16	6	0	-0.854782	-4.035832	-0.522562
17	6	0	1.853393	3.605058	0.137420
18	6	0	0.257418	1.831512	-0.080666
19	6	0	-1.798108	-2.906972	-0.895482
20	6	0	0.818793	4.505290	0.330097
21	6	0	-0.764152	2.741620	0.124281
22	6	0	-2.525995	-2.271518	0.250509
23	6	0	-0.501579	4.087318	0.331018
24	6	0	-3.127852	-0.926506	0.053727
25	6	0	-3.149239	-0.296588	-1.191936
26	6	0	-3.730140	-0.291444	1.141060
27	6	0	-3.769260	0.928431	-1.356262
28	6	0	-4.343656	0.936623	0.997413
29	6	0	-4.354088	1.516334	-0.255289
30	1	0	0.978390	-0.242766	-1.541623
31	1	0	2.530359	-0.750544	-2.180976
32	1	0	1.176549	0.104001	1.469802
33	1	0	2.809122	-0.247049	2.015694
34	1	0	2.405568	-2.880419	-0.969156
35	1	0	0.884820	-2.598161	-1.814199
36	1	0	1.167731	-2.129875	2.301383
37	1	0	2.577664	-2.606786	1.346778
38	1	0	4.049687	2.251868	1.048410
39	1	0	4.327489	2.633944	-0.666417
40	1	0	5.803591	0.672828	-0.019099
41	1	0	1.037360	-4.418441	0.435147
42	1	0	-0.168853	-3.678390	1.488221
43	1	0	-1.413939	-4.877456	-0.100490
44	1	0	-0.406893	-4.408211	-1.450488
45	1	0	2.869738	3.981357	0.141924
46	1	0	-0.014382	0.796563	-0.243507
47	1	0	-1.268349	-2.132996	-1.459178
48	1	0	-2.573259	-3.281310	-1.580331
49	1	0	1.059907	5.552739	0.487170
50	1	0	-1.788999	2.378516	0.123378
51	1	0	-1.309155	4.794213	0.489959
52	1	0	-2.694507	-0.764910	-2.057772
53	1	0	-3.715267	-0.775203	2.111748
54	1	0	-3.801914	1.427562	-2.318455
55	1	0	-4.812580	1.445409	1.832582

----

chlorprothixene

Input orientation:

Center Number	Atomic Number	Atomic Type	Coordinates (Angstroms) X	Y	Z

1	17	0	-3.991448	3.310975	1.290303
2	16	0	-1.729637	-1.730460	-1.669725
3	7	0	4.487478	1.654080	-0.535418
4	6	0	-0.096036	0.030888	0.241828
5	6	0	2.330080	0.793972	0.387855
6	6	0	-1.508369	0.416358	0.057199
7	6	0	0.166715	-1.417548	0.320899
8	6	0	0.855756	0.973015	0.291222
9	6	0	3.043081	1.833916	-0.456654
10	6	0	-2.338780	-0.316803	-0.794291
11	6	0	-0.517337	-2.307523	-0.512380
12	6	0	-2.044450	1.525641	0.708528
13	6	0	1.075893	-1.952075	1.234963
14	6	0	-3.652068	0.075945	-1.021235
15	6	0	-0.249847	-3.670585	-0.480170
16	6	0	-3.348516	1.904460	0.466523
17	6	0	1.343524	-3.309011	1.273783
18	6	0	5.023070	2.665032	-1.431844
19	6	0	5.091989	1.836494	0.774417
20	6	0	-4.164651	1.199212	-0.399684
21	6	0	0.691911	-4.169285	0.401966
22	1	0	2.625314	-0.213603	0.072897
23	1	0	2.639553	0.892504	1.437785
24	1	0	2.643936	1.798114	-1.477855
25	1	0	2.806845	2.843488	-0.062320
26	1	0	0.527521	2.010363	0.206159
27	1	0	-1.433353	2.090613	1.405329
28	1	0	1.572749	-1.288199	1.935560
29	1	0	-4.283547	-0.503200	-1.688673
30	1	0	-0.791153	-4.341601	-1.141340
31	1	0	2.059687	-3.697190	1.990748
32	1	0	4.812858	3.687814	-1.068964
33	1	0	4.582715	2.562471	-2.427397
34	1	0	6.106949	2.555347	-1.521860
35	1	0	4.817690	2.812071	1.216910
36	1	0	6.180820	1.798244	0.686736
37	1	0	4.786507	1.048659	1.466217
38	1	0	-5.189916	1.508401	-0.570387
39	1	0	0.897164	-5.234793	0.424973

----

raclopride

Input orientation:

Center	Atomic	Atomic	Coordinates (Angstroms)		
Number	Number	Type	X	Y	Z

1	17	0	4.124728	2.422160	0.892064
2	17	0	4.913477	-2.274556	-1.612153
3	8	0	-0.267755	-1.901359	-0.607322
4	8	0	1.341108	1.533033	1.072311
5	8	0	2.073790	-2.635641	-1.226377
6	7	0	-4.159056	-0.205512	0.095631
7	7	0	-0.549259	-0.411491	1.051416
8	6	0	-2.756861	0.221897	0.181261
9	6	0	-2.792735	1.702333	0.589397
10	6	0	-4.276798	2.073763	0.579167
11	6	0	-4.885420	1.001216	-0.293718
12	6	0	-1.976782	-0.653376	1.144978
13	6	0	-4.317179	-1.286546	-0.868874
14	6	0	-5.728639	-1.813206	-0.956985
15	6	0	0.194926	-0.992413	0.107688
16	6	0	1.621395	-0.585201	-0.043371
17	6	0	2.140154	0.629453	0.435295
18	6	0	2.488265	-1.478712	-0.707064
19	6	0	3.487106	0.919506	0.284353
20	6	0	3.841317	-1.154699	-0.822086
21	6	0	4.342528	0.027030	-0.332704
22	6	0	0.765975	2.504380	0.190098
23	1	0	-2.287811	0.134918	-0.818351
24	1	0	-2.231108	2.306297	-0.128612
25	1	0	-2.331562	1.861241	1.570451
26	1	0	-4.699135	2.013192	1.587554
27	1	0	-4.463583	3.081040	0.200646
28	1	0	-5.963036	0.883347	-0.153264
29	1	0	-4.706615	1.227068	-1.363947
30	1	0	-2.164906	-1.712037	0.953372
31	1	0	-2.289367	-0.439378	2.171328
32	1	0	-3.987313	-0.938834	-1.867536
33	1	0	-3.639139	-2.100509	-0.586614
34	1	0	-0.157468	0.396351	1.523971
35	1	0	-6.087745	-2.154874	0.019196
36	1	0	-6.423823	-1.054160	-1.328163
37	1	0	-5.770590	-2.661212	-1.646183
38	1	0	5.396598	0.260446	-0.437168
39	1	0	1.084313	-2.639108	-1.140707
40	1	0	0.091922	2.019485	-0.525909
41	1	0	1.549249	3.043729	-0.351243
42	1	0	0.206534	3.200938	0.814275

----

sulpiride

Input orientation:

Center Number	Atomic Number	Atomic Type	Coordinates (Angstroms) X	Y	Z
1	16	0	4.881157	1.048770	0.736578
2	8	0	-0.307039	1.662060	0.409487
3	8	0	0.250977	-2.108843	-0.982908
4	8	0	4.523734	1.765869	1.941810
5	8	0	6.045523	0.189979	0.772492
6	7	0	-4.650507	0.486816	-0.112319
7	7	0	-1.064610	0.263821	-1.194606
8	7	0	4.997091	2.201400	-0.432582
9	6	0	-3.267513	0.008829	-0.173949
10	6	0	-3.393622	-1.477467	-0.496817
11	6	0	-4.788703	-1.865436	0.014214
12	6	0	-5.332180	-0.582543	0.619024
13	6	0	-2.424500	0.768184	-1.177557
14	6	0	-4.743739	1.763711	0.586334
15	6	0	-6.155182	2.286498	0.700238
16	6	0	-0.139284	0.679373	-0.315352
17	6	0	1.164114	-0.057419	-0.282842
18	6	0	2.266019	0.668349	0.127945
19	6	0	1.339620	-1.419705	-0.609781
20	6	0	3.518344	0.081157	0.195664
21	6	0	2.600782	-2.002659	-0.522596
22	6	0	3.690459	-1.252920	-0.127120
23	6	0	0.376002	-3.490514	-1.296677
24	1	0	-2.800044	0.122487	0.826381
25	1	0	-3.315466	-1.633064	-1.579883
26	1	0	-2.590621	-2.055007	-0.030853
27	1	0	-4.762504	-2.672972	0.749081
28	1	0	-5.422851	-2.199340	-0.811585
29	1	0	-5.071981	-0.521785	1.693696
30	1	0	-6.418046	-0.494912	0.537508
31	1	0	-2.394970	1.836575	-0.955990
32	1	0	-2.847173	0.644014	-2.179764
33	1	0	-4.131128	2.497177	0.051614
34	1	0	-4.302354	1.660493	1.597264
35	1	0	-0.911205	-0.634545	-1.636997
36	1	0	-6.149758	3.278450	1.160537
37	1	0	-6.783506	1.640540	1.320288
38	1	0	-6.626303	2.376317	-0.284160
39	1	0	2.129271	1.713013	0.390005
40	1	0	2.738550	-3.048197	-0.766499
41	1	0	4.671372	-1.711284	-0.069235
42	1	0	5.513163	1.864436	-1.243855
43	1	0	5.428070	3.042395	-0.050208

44	1	0	-0.625982	-3.824837	-1.562298
45	1	0	1.050199	-3.639304	-2.144775
46	1	0	0.732427	-4.055063	-0.430652

----

clopenthixol

Standard orientation:

Center Number	Atomic Number	Atomic Type	Coordinates (Angstroms) X	Y	Z
1	17	0	4.897079	-4.005970	-0.969200
2	16	0	3.926331	1.670150	1.509388
3	8	0	-9.328891	-0.291098	-0.520054
4	7	0	-2.904811	-0.152894	0.533173
5	7	0	-5.751395	-0.732048	0.400205
6	6	0	-3.569460	-0.529756	-0.710284
7	6	0	-3.634687	-0.767767	1.636695
8	6	0	-5.021523	-0.126343	-0.710716
9	6	0	-5.080971	-0.354260	1.640816
10	6	0	-1.531795	-0.635891	0.553373
11	6	0	-7.121526	-0.245769	0.446485
12	6	0	-0.612496	0.056489	-0.437582
13	6	0	-7.987578	-0.722896	-0.695765
14	6	0	0.773716	-0.465640	-0.298260
15	6	0	1.929549	0.212648	-0.306693
16	6	0	3.199481	-0.493688	-0.049302
17	6	0	2.038710	1.667770	-0.513946
18	6	0	4.174274	0.079516	0.771664
19	6	0	2.913720	2.428434	0.267026
20	6	0	3.450810	-1.751007	-0.594856
21	6	0	1.304598	2.328125	-1.499875
22	6	0	5.342180	-0.608397	1.077178
23	6	0	2.997924	3.806711	0.113325
24	6	0	4.614436	-2.420818	-0.277547
25	6	0	1.386489	3.700147	-1.658837
26	6	0	5.565890	-1.871899	0.563212
27	6	0	2.222995	4.444024	-0.838937
28	1	0	-3.488718	-1.623720	-0.867200
29	1	0	-3.075110	-0.043777	-1.556114
30	1	0	-3.168062	-0.470261	2.581445
31	1	0	-3.567455	-1.871462	1.572147
32	1	0	-5.098953	0.977457	-0.657749
33	1	0	-5.467050	-0.439300	-1.658686
34	1	0	-5.594593	-0.831643	2.481664
35	1	0	-5.150174	0.740389	1.794022
36	1	0	-1.507293	-1.728853	0.370523

37	1	0	-1.138269	-0.483029	1.566055
38	1	0	-7.141138	0.862885	0.470170
39	1	0	-7.575753	-0.589812	1.383310
40	1	0	-0.966670	-0.122733	-1.462716
41	1	0	-0.646823	1.140325	-0.281782
42	1	0	-8.012425	-1.816829	-0.721597
43	1	0	-7.605973	-0.376314	-1.663952
44	1	0	0.836174	-1.539711	-0.115584
45	1	0	-9.342505	0.671220	-0.583165
46	1	0	2.728899	-2.202474	-1.268218
47	1	0	0.666290	1.747419	-2.158670
48	1	0	6.088449	-0.151867	1.720779
49	1	0	3.681979	4.377669	0.734890
50	1	0	0.800378	4.190043	-2.429582
51	1	0	6.476680	-2.412136	0.796981
52	1	0	2.292650	5.520577	-0.957042
