# Supplementary material for: A quantum chemical approach representing a new perspective concerning agonist and antagonist drugs in the context of schizophrenia and Parkinson’s disease
Source: PLoS One. 2019 Dec 12;14(12):e0224691. doi: 10.1371/journal.pone.0224691 (PMC6907805; doi:10.1371/journal.pone.0224691)
Supplement: Attachment — Submitted filename: letterMARTINEZplosONEresponse1.docx [file pone.0224691.s002.docx]

PONE-D-19-20849
A quantum chemistry approach representing a new perspective concerning agonist and antagonist drugs in the context of schizophrenia and Parkinson’s disease

Professor Dennis R. Salahub

Academic Editor

PLOS ONE

Dear Prof. Salahub,

We thank the reviewers for their priceless evaluations and their very useful comments. Since both reviewers have raised the issue of bringing in the properties of the receptors, we would like to start explaining why we do not include the properties of the receptors.

In this investigation we provide a classification of thirty antipsychotics in terms of their electron donor-acceptor properties that give information about the intrinsic reactivity of these molecules. These characteristics of the drugs are independent of the receptors, and allow us to have a clear and straightforward classification of the antipsychotics, which, otherwise, would not be possible to achieve. Currently, a way to classify the antipsychotics is based on *In* *Vivo Rotational Experiments* with injured rats. In one of these reports, “a lesion on rats was performed on the left side of the medial forebrain bundle in the brain” and the conclusion is that “the rotations produced upon agonist Challenger were clockwise”. These experiments are extremely impressive with conclusions that are based on the behavior of four rats that have been injured. We believe that with our quantum chemical calculations, it is possible to have another classification and both classifications can complement each other.

The study of the receptors is a matter of a different investigation and in fact, there are previous studies that predict the binding sites and report binding energies of these antipsychotics with dopamine receptors (see for example references 46 and 53 of the manuscript). In spite of all the studies concerning the drugs and the receptors reported until now, there are not investigations regarding the intrinsic characteristics of these drugs. We strongly believe that it is still needed to be explained why some drugs behave as agonists and others as antagonists from the quantum chemistry point of view, and the intrinsic characteristics that we report here could give some solid ideas.

The intrinsic characteristics of these drugs allow us to classify them and also give us some ideas about possible reaction mechanisms, but it is clear that, to speak about the reaction mechanisms it is important to consider all the reactants, in this case, drugs and receptors. In this sense, the paper in the original version was confusing since we included a possible reaction mechanism and, as both reviewers established, it is not possible to analyze a reaction mechanism with only one of the reactants. In that case, the study of the GPCR proteins is also necessary. What we can do is a clear classification. For this reason, we removed, from the original version, all the information concerning the reaction mechanism, including Figure 4 and 6. Specifically, the following paragraphs were deleted in this new version:

*We assume that the receptors (GPCR proteins) are represented by the electron bath and, in this context; they are able to either donate charge to the drugs or accept charge from them.*

*It is known that these drugs interact with GPCR through ligand-receptor non-covalent interactions but what happens once the drug is bonded to the receptor? Our premise is that once the drug interacts with the receptor, a charge-transfer process initiates. Dopamine binds to the receptors and activates them, and some of this activation may be the result of electron transfer; if this is the case, dopamine will donate electrons to the receptors.*

*… and therefore the may also transfer electrons to the receptors and activate them*

*… would accept electrons form the receptors, impeding activation.*

*The model presented in here gives prominence to the charge transfer process in the action mechanism: drugs interact with the receptors and a charge transfer occurs. Dopamine activates the receptors by donating electrons to GPCRs. Agonists also transfer electrons to the receptors and activated them, as they have similar electron donor capacity. Conversely, the antagonists accept electrons from the receptors, so activation does not proceed.*

Figure 4. Schematic representation of the action mechanisms proposed in this report. Dopamine and agonist are good electron donors (represented by a red hexagon) and therefore, transfer electrons to the receptor. Antagonists (represented as a blue hexagon) are good electron acceptors and may accept electrons from the receptor.

Figure 6. Schematic representation of the action mechanism proposed in this report. Dopamine and agonist are hard molecules and good electron donors (represented by a red hexagon) and therefore they transfer electrons to the receptor without polarization. Antagonists (represented as a blue deformed hexagon) are soft molecules and good electron acceptors; they are polarizable and may accept electrons from the receptor.

Finally, in here we present and explain other modifications that were carried out on the manuscript. We hope to have been able to solve and properly answered the reviewers' concerns.

With my best regards

Prof. Ana Martínez

**Reviewer #1:**

1. The author should describe the dopaminergic pathway details in the introduction part.

**Authors’ reply**

Following this suggestion, the first paragraph of the introduction was modified as follows:

*Schizophrenia, a type of psychosis, is associated with diverse symptoms such as avolition, catatonia, diminished emotional expression, anhedonia, disorganized speech, delusions, hallucinations and psychomotor abnormality [1-10]. The main hypothesis that could explain schizophrenia is related to the neurotransmitter dopamine. In the dopaminergic system dopamine is synthesized in dopaminergic nerve terminals from the amino acid tyrosine. Then it is absorbed by a vesicular monoamine transporter where is stored until it is used during neurotransmission, which is regulated by the receptors. The dopamine hypothesis of schizophrenia states that the physiological mechanism evolves from an excess of dopamine activity in certain regions of the brain and little dopamine activity in other regions. Several drugs named antipsychotics have been developed to control the symptoms of schizophrenia, which primarily target this dopaminergic pathway [11-37].*

**Reviewer #1:**

2. In the ligand-receptor interaction the authors, should describe about the ligand-receptor non-covalent interactions. Further, the drug is bonded to the receptor?

**Authors’ reply**

We thank the reviewer for this comment that is very interesting. However, with the results reported here it is not possible to analyze the ligand-receptor interaction since the receptor is not included in this investigation. We are focused on the analysis of the drugs.

**Reviewer #1:**

3. The electron bath is unclear in the receptors (GPCR proteins). The authors should describe the electron transfer bath and provide the image.

**Authors’ reply**

As we explain before in this letter, we are not including the GPCR proteins in this study.

**Reviewer #1:**

4. The authors claimed, the dopamine electron transfer properties are similar to this neurotransmitter but the authors did not describe about the similarities.

**Authors’ reply**

We apologize for the lack of clarity in the explanation. We added the following paragraph

*As can be seen in Figure 2, agonists are located close to dopamine in the DAM. This means that they are more like dopamine than antagonists. i.e. they are good electron donors being the best quinpirole.*

**Reviewer #1:**

5. The authors should provide the clear image about the “Agonists have similar electron donor capacity and therefore they may also transfer electrons to the receptors and activate them. Contrastingly, antagonists would accept electrons from the receptors, impeding activation”.

**Authors’ reply**

As we pointed out before, in this new version we removed all the information concerning the reaction mechanism.

**Reviewer #2:**

The authors said that they are not talking about receptor. Receptors are not receptor-like in fig 4, but are receptors and family of receptors. Each molecule binds on several receptors, which are identified or even sometimes not identified. Analysis of sequences of different receptors reveals that the active site of this GPCR family is kite different. The activity of the molecule if the binding part of the mechanism is well-known this some crystallographic structure, the mechanism of the biologic response is unknown. In conclusion of this remark, an analysis of site in term of residues should give a validation if site is fill with residues acceptor of electrons, an electron donor molecule should have a good binding.

**Authors’ reply**

As we already explained previously, the main objective of this investigation is to classify the antipsychotics by using intrinsic properties. It is a good idea to include residues in order to analyze the binding scheme, but this is a matter of a different investigation. The classification presented here could be very useful for future investigations and also in the clinic of these patients.

**Reviewer #2:**

The list of molecules studied is concise in the article. First, second, and third generations of antipsychotic drugs and agonists are more numerous than in the list of molecules. A full list is necessary to validate the assumption of agonist or antagonist drugs is correlated this the acceptor or donor of electrons. The case is more complicated than a molecule has nitrogen atom or halogen it is antagonism, and a molecule has a hydroxy function; it is an agonist.

**Authors’ reply**

We agree with the reviewer. There are hundreds of antipsychotics, but the drugs presented here are the ones that have been used for several years and they are very well characterized. Other drugs are not completely characterized, and others bind to several receptors, or are agonists/antagonist of other neurotransmitters. For some of these thirty antipsychotics there are theoretical studies concerning the binding site to the dopamine receptor. At this point we do not generalize the properties for agonists/antagonists but we classify these 30 molecules according to the electron/donor-acceptor properties.
