# Supplementary material for: A quantum chemical approach representing a new perspective concerning agonist and antagonist drugs in the context of schizophrenia and Parkinson’s disease
Source: PLoS One. 2019 Dec 12;14(12):e0224691. doi: 10.1371/journal.pone.0224691 (PMC6907805; doi:10.1371/journal.pone.0224691)
Supplement: Attachment — Submitted filename: letterMARTINEZplosONEresponse2.docx [file pone.0224691.s003.docx]

PONE-D-19-20849
A quantum chemistry approach representing a new perspective concerning agonist and antagonist drugs in the context of schizophrenia and Parkinson’s disease

Professor Dennis R. Salahub

Academic Editor

PLOS ONE

Dear Prof. Salahub,

We thank the useful comments of both reviewers. Reviewer 2 requests the justification for the choice of molecules. We revised the manuscript accordingly, and we added the following paragraphs at the end of the **Methods** section.

*In this investigation we analyze 20 antagonists and 10 agonists of dopamine (see Figure 1). These molecules have been selected due to the following reasons [11]. The selected First Generation Antipsychotics represent different families of compounds. Haloperidol, trifluperidol, benperidol, spiperone, pipamperone and droperidol are typical antipsychotic of the butyrophenone family. They exhibit high affinity dopamine D2 receptor antagonism and slow receptor dissociation kinetics. These butyrophenones have different receptor binding profiles and exhibited distinctive clinical efficacy. Haloperidol is one of the first drugs used to treat schizophrenia and it is the most commonly used. Trifluperidol is stronger than haloperidol. Benperidol and spiperone are two of the most potent antipsychotics in this family. Moreover, spiperone is used in treating drug-resistant schizophrenia. Pipamperone present a different pharmacological profile and it can be classified as a first-generation typical antipsychotic. It was considered as a forerunner of atypical antipsychotics. Chlorprothixene and clopenthixol are typical antipsychotics of the thioxanthene group. Raclopride is a selective antagonist of dopamine receptors and it can be radiolabelled and used as a tracer for in vitro imaging. Sulpiride belongs to the benzamide class. Within this group of molecules, we can compare ligands of the same family and also ligands from different families.*

*The First Generation Antipsychotics have largely given way to Second Generation Antipsychotics such as risperidone, clozapine, olanzapine, quetiapine and ziprasidone. Clozapine was the first antipsychotic of the second generation that was synthesized and tested. It has antipsychotic action but no Parkinson-like motor side effects and it is one of the most widely used together with risperidone. Clozapine is the most efficacious antipsychotic drug and it is used only for treatment of resistant schizophrenia due to its severe side effects. Olanzapine has a similar structure and it is also a good antipsychotic but with lower side effects. Risperidone, the second atypical antipsychotic quickly became a first-line treatment for acute and chronic schizophrenia because of its preferential side effect profile. Ziprasidone was also developed based upon clozapine and it is used since 1998. All these antipsychotics are widely used in the treatment of schizophrenia.*

*Aripiprazole and cariprazine are relatively new antipsychotic drugs and represents the Third Generation of Antipsychotics. They are dopamine system stabilizers. Finally, the agonists that we investigated were selective developed to be agonists and therefore, they are very well characterized as agonists. These molecules were investigated theoretically with docking studies that allow investigating potential sites of interaction [53].*

We hope to have been able to solve and properly answered the reviewer's concerns.

Finally, we kindly would like to request if possible, a discount on the fees of this open access. Unfortunately, we do not have any funding (grants) that can cover these fees and therefore, we should pay this contribution with our own money.

With my best regards

Prof. Ana Martínez
